# Identification of the relationship between Chinese *Adiantum reniforme* var. *sinense* and Canary *Adiantum reniforme*

**DOI:** 10.1186/s12870-014-0361-9

**Published:** 2015-02-05

**Authors:** Ai-Hua Wang, Ye Sun, Harald Schneider, Jun-Wen Zhai, Dong-Ming Liu, Jin-Song Zhou, Fu-Wu Xing, Hong-Feng Chen, Fa-Guo Wang

**Affiliations:** Key Laboratory of Plant Resources Conservation and Sustainable Utilization, South China Botanical Garden, Chinese Academy of Sciences, Guangzhou, 510650 China; University of Chinese Academy of Sciences, Beijing, 100049 China; Department of Life Sciences, Natural History Museum, London, SW75BD UK; College of Landscape Architecture, Fujian Agriculture and Forestry University, Fuzhou, 350002 China; College of Chinese Traditional Medicine, Guangzhou University of Chinese Medicine, Guangzhou, 510006 China

**Keywords:** Chromosome numbers, cpDNA, Flow cytometry, Molecular clock dating, Morphological characters, Phylogenetic position, Relationship identification, SEM observation

## Abstract

**Background:**

There are different opinions about the relationship of two disjunctively distributed varieties *Adiantum reniforme* L. var. *sinense* Y.X.Lin and *Adiantum reniforme* L. *Adiantum reniforme* var. *sinense* is an endangered fern only distributed in a narrowed region of Chongqing city in China, while *Adiantum reniforme* var. *reniforme* just distributed in Canary Islands and Madeira off the north-western African coast. To verify the relationship of these two taxa, relative phylogenetic analyses, karyotype analyses, microscopic spore observations and morphological studies were performed in this study. Besides, divergence time between *A. reniforme* var. *sinense* and *A. reniforme* var. *reniforme* was estimated using GTR model according to a phylogeny tree constructed with the three cpDNA markers *atp*A, *atp*B, and *rbc*L.

**Results:**

Phylogenetic results and divergence time analyses--all individuals of *A. reniforme* var. *sinense* from 4 different populations (representing all biogeographic distributions) were clustered into one clade and all individuals of *A. reniforme* var. *reniforme* from 7 different populations (all biogeographic distributions are included) were clustered into another clade. The divergence between *A. reniforme* var. *reniforme* and *A. reniforme* var. *sinense* was estimated to be 4.94 (2.26-8.66) Myr. Based on karyotype analyses, *A. reniforme* var. *reniforme* was deduced to be hexaploidy with 2n = 180, X = 30, while *A. reniforme* var. *sinense* was known as tetraploidy. Microscopic spore observations suggested that surface ornamentation of *A. reniforme* var. *reniforme* is psilate, but that of *A. reniforme* var*. sinense* is rugate. Leaf blades of *A. reniforme* var. *sinense* are membranous and reniform and with several obvious concentric rings, and leaves of *A. reniforme* var. *reniforme* are pachyphyllous and coriaceous and are much rounder and similar to palm.

**Conclusion:**

*Adiantum reniforme* var. *sinense* is an independent species rather than the variety of *Adiantum reniforme* var. *reniforme*. As a result, we approve *Adiantum nelumboides* X. C. Zhang, nom. & stat. nov. as a legal name instead of the former *Adiantum reniforme* var. *sinense*. China was determined to be the most probable evolution centre based on the results of phylogenetic analyses, divergence estimation, relative palaeogeography and palaeoclimate materials.

**Electronic supplementary material:**

The online version of this article (doi:10.1186/s12870-014-0361-9) contains supplementary material, which is available to authorized users.

## Background

*Adiantum reniforme* L. var. *sinense* Y.X.Lin (Chinese name “He ye jin qian cao”) was first discovered in Chongqing city in China in 1978 [1]. It was published in *Acta Phytotaxonomica Sinica* as a variety of *Adiantum reniforme* L. because of their similar morphological characters in 1980. It is only distributed along the Yangtze River from Shizhu County to the Wanzhou District of Chongqing, which stretches for almost 100 kilometres through Xi-tuo, Xin-xiang, Wu-ling, Chang-ping and other places [2-4]. It has a narrow distribution zone and an endangered status. *A. reniforme* var. *sinense* was listed as a class II protected fern in China [2]. The plant is known to have medicinal uses including heat-clearing and detoxifying, promoting diuresis and relieving stranguria, curing icteric hepatitis and stones [5]. As a result, the plant has been over-collected by local people. Additionally, the construction of the Three Gorges Dam from 1993 to 2009 caused destruction of habitats and reduced its population size, which reduced gene flow among populations [6]. Many studies have been conducted to protect *A. reniforme* var. *sinense* from extinction. These studies included field habitat investigations [2], the use of spore propagation technology [7] and increases in population gene diversity [6,8,9]. *A. reniforme* var. *sinense* was previously shown to be tetraploid (2n = 120, X = 30) in Lin YX [10]. Scanning electron microscopy (SEM) analysis of *A. reniforme* var. *sinense* suggested that its spores are actinomorphic and trilete with polar surface triangles. Additionally, the equatorial surface is semicircular or super-semicircular, and the surface ornamentation is psilate [11]. *Adiantum* belongs to the family Pteridaceae, although different opinions exist regarding whether *Adiantum* is monophyletic or paraphyletic with vittarioid ferns [12-17]. A phylogenetic tree of Chinese *Adiantum* was constructed using five cpDNA primers for the following genes: *atp*A, *atp*B, *rbc*L, *trn*L-F and *trn*S. This analysis indicated that *Adiantum* was monophyletic and *A. reniforme* var. *sinense* was closely related to *Adiantum* Ser. *Venusta,* which was established by Ching Renchang in *Flora Republicae Popularis Sinicae*, *Tomus* 3(1) [18].

There are a limited number of reports of *A. reniforme* var. *reniforme*. The first specimens were collected in Madeira, and it was first published in *Species Plantarum* by Linnaeus in 1753. The plant is found in the Canary Islands and Madeira off the north-western African coast. Manton [19] considered *A. reniforme* var. *reniforme* as decaploid (2n = 300, X = 30) after her study on the specimens kept in Kew garden but collected in Madeira and Tenerife. In 1985, Mary Gibby restudied ploidy and the chromosomes of materials collected in the Canary Island and suggested that it was tetraploid (2n = 120, X = 30). However, there is no photographic record of this result. Subsequent studies have demonstrated that ploidy levels of all ferns in the Canary Islands are no more than hexaploid [20]. Consequently, the ploidy of *A. reniforme* var. *reniforme* is controversial, and the differences in chromosome number between the Canary population and the Madeira population are unclear.

**Figure 1:**
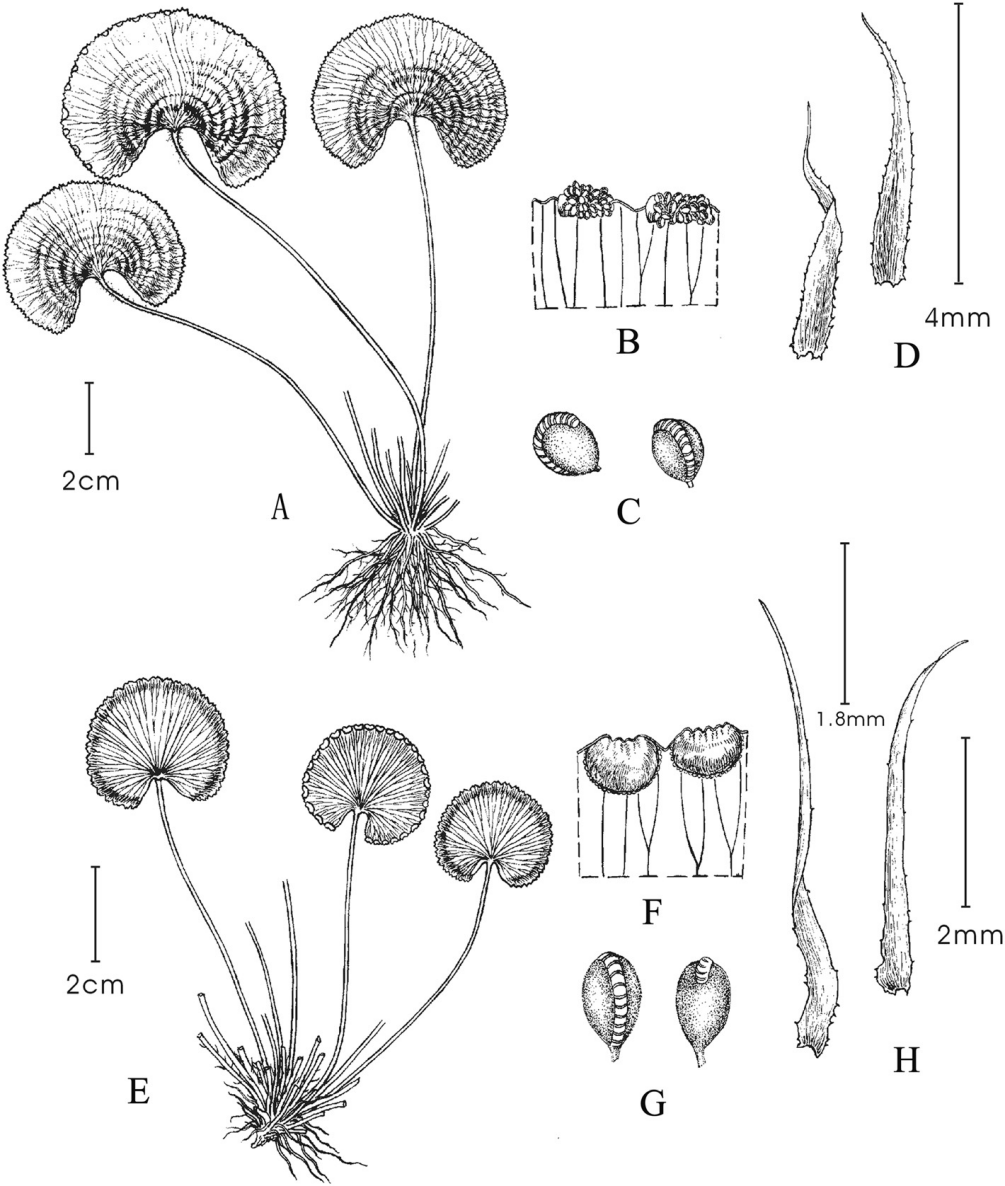
**Morphological characters of** ***A. reniforme*** **var.** ***sinense*** **and** ***A. reniforme*** **var.** ***reniforme*** **. A**, **B**, **C**, and **D** represent the leaf, sporangiorus, sporangium and scales of A. *reniforme* var. sinense, respectively. **E**, **F**, **G**, and **H** represent the related leaf, sporangiorus, sporangium and scales of A. *reniforme* var. sinense, respectively.

There are similar morphological characters between *A. reniforme* var. *sinense* and *A. reniforme* var. *reniforme*. So, it seems reasonable that they are varieties. However, the China-Canary distribution disjunction of these two taxa makes their relationships doubtful. Zhang XC [21] treated *A. reniforme* var. *reniforme* as an independent species in the book “*Lycophytes and ferns of China*” but without explanation. As described above, the spore morphology, karyotype analysis and phylogenetic analysis of *A. reniforme* var. *reniforme* are currently unknown. Because of the limited morphological characters of these two taxa, for example, only one single leaf blade with one petiole, it is not convictive for the treatment that they were varieties between each other just based on their limited morphological characters (see Figure 1). Additional studies are required to determine whether *A. reniforme* var. *sinense* is a variety or an independent species. To make the taxonomy relationship between *A. reniforme* var. *sinense* and *A. reniforme* var. *reniforme* clear and deduce mechanisms of the intercontinental disjunction, we have analysed 7 populations consisting of almost 96 individuals of *A. reniforme* var. *sinense* from China and 8 populations consisting of almost 164 individuals of *A. reniforme* var. *reniforme* from Canary and Madeira.

## Methods

### Materials

In this study, 24 individuals from 11 populations of both the *Adiantum reniforme* var. *reniforme* and *A. reniforme* var. *sinense* representing all biogeographic distributions were sampled and sequenced. The 31 species of *Adiantum* and *Vittaria flexuosa* (outgroup) were downloaded from GenBank to construct a phylogeny tree of *Adiantum* with the combined cpDNA markers *atp*A, *atp*B, *trn*L-F and *trn*S. Furthermore, three plastid genes (*rbc*L, *atp*A, and *atp*B) from 24 outgroup species were downloaded to test the divergence time of *Adiantum reniforme* var. *reniforme* and *A. reniforme* var. *sinense*. All taxa included in this study, voucher information and collection sites are listed in Additional file 1 and Addition file 2.

### DNA extraction, amplification and sequencing

Total DNA was extracted from 20 mg silica-gel-dried leaf material using a modified CTAB DNA extraction protocol [22]. The *atp*A gene was amplified with primers “ESATPF412F”and“ESTRNR46F” [23]. “ESATB172F” and “ESATPE45R” were used for amplifying and sequencing the *atp*B gene [14]. “1 F” and “1379R” were used to amplify and sequence the *rbc*L gene [24]. The *trn*L-F region was amplified and sequenced with primers “p1” and “f” [25,26]. Primers “*trn*S” [27] and “*rps*4.5” [28] were used to amplify and sequence the *rps*4*-trn*S region. All amplifications were performed in a 30-μL reaction mixture. The PCR reactions contained the following reagents: 1.0-2.4 μL of each primer (5p), 17-60 ng sample DNA, 1.5 U of Taq DNA polymerase, 10 × buffer (including Mg^2+^), 0.25 mmol · L^-1^dNTP, and ultrapure water (ddH_2_O). The *atp*A and *atp*B 30-μL reaction mixtures were incubated at 95°C for 10 min, cycled 35 times (95°C for 1 min, 50°C for 1 min, and 72°C for 100 s), followed by a final extension for 10 min at 72°C. The *rbc*L and *trn*L-F PCR reactions were incubated at 95°C for 3 min, cycled 35 times (95°C for 1 min, 51°C for 1 min, and 72°C for 80 s), followed by a final extension for 10 min at 72°C. The *rps*4-*trn*S PCR reactions were incubated at 95°C for 3 min, cycled 35 times (94°C for 30 s, 58°C for 45 s, and 72°C for 80 s), followed by a final extension for 10 min at 72°C. The PCR products were purified and sequenced with an ABI 3730XL by Majorbio Company.

### Phylogenetic analyses

The sequences were assembled with Sequencher 4.14 and then adjusted manually through Bioedit v.7.1.3 [29] and aligned using the program Clustal X version 2.0 [30]. Phylogenetic trees of each individual and the combined markers (*atp*A, *atp*B, *rbc*L, *trn*L-F, and *rps*4-*trn*S) were constructed using maximum parsimony (MP) and Bayesian Markov chain Monte Carlo (MCMC) inference. The maximum parsimony analyses were performed with PAUP* 4.0b10 [31], treating gaps as missing data and using the heuristic search options with 1000 random replicates and tree-bisection-reconnection (TBR) branch swapping. All characteristics were unordered and equally weighted. For Bayesian analyses, MrModeltest2 (v2.3; [32]) based on the Akaike information criterion (AIC) was used to identify the best-fit molecular evolution model for each of the DNA markers. We constructed Bayesian trees using MrBayes 3.1 [33] with the best-fit model GTR + I + G. Trees were generated for 1,000,000 generations, sampling every 100 generations. Four chains were used with a random initial tree. For each of the individual data partitions and the combined dataset, the first 2500 sample trees were discarded as burn-in to ensure that the chains reached stationarity. Nodes receiving bootstrap support (BS) of < 70% in the MP analyses or PP of < 0.95 in the BI analyses were not considered to be well supported.

### Molecular clock dating

Bayesian molecular dating studies were performed with the combined dataset of *rbc*L, *atp*A and *atp*B. Sequences of 24 outgroup species were downloaded from NCBI. The divergence time estimation of each clade in *Adiantum* and their credibility intervals were implemented in BEAUTI ⁄ BEAST 1.7.4 [34]. The BEAST analyses were performed with the GTR model, the uncorrelated relaxed lognormal clock model and the coalescent exponential growth tree. We used the 65.5 ± 0.3 Myr, which was the crown of the ceratopteridoids clade [35], as the calibration point. Posterior distributions of parameters were approximated using three independent MCMC analyses of 20,000,000 generations with 10% burn-in. Convergence was examined using Tracer 1.5 [36].

### Karyotype analysis

To deduce the ploidy levels of *A. reniforme* var. *reniforme*, *A. reniforme* var. *sinense* was used as an internal standard because of its clear sporophytic chromosomes (2n = 120, X = 30), as displayed in Lin YX [10]. There were 32 sporophytic materials from different populations of both taxa examined by flow cytometric analyses to confirm the accuracy of ploidy levels for *A. reniforme* var. *reniforme* (Table 1). The leaves have membranous and hard leaf blades, so young and fresh blades spreading from circinate leaves were used. Small pieces of plant leaves were chopped with a double-edged razor in a Petri dish containing 0.4 mL mixed buffer (including ice-cold Otto buffer combined with DAPI fluorochrome, as patented by Partec Comneruim). Then, an additional 1.6 mL of buffer was mixed with the cells in the Petri dish and the cells were filtered through a 30-μm-mesh filter into a 5-mL cytometry tube. The tube was incubated in the dark at room temperature for 5-10 min. Each sample was analysed on a flow cytometer (Cyflow Space, Partec) equipped with a high-pressure mercury arc lamp for UV excitation. For each sample, a minimum of 2,000 nuclei were analysed. The fluorescence peaks and relative fluorescence intensity were analysed by the software Flomax.

**Table 1:** **Relative fluorescence intensity (DAPI measurements) for the** ***A. reniforme*** **var.** ***sinense*** **and** ***A. reniforme*** **var.** ***reniforme*** **, summarised by the phytogeographic regions**

**Taxon**	**Ploidy level**	**Accession number**	**Region**	**Relative fluorescence intensity**	**Relative fluorescence intensity (mean ± s.d.)**	**Overall mean (±s.d.)**
*A.reniforme* var. *sinense*	4X	WAH009	xi-tuo, shi zhu, China	62.06	65.44 ± 3.59	65.44 ± 3.59
		WAH007	xi-tuo, shi zhu, China	65.06		
		WAH003	xi-tuo, shi zhu, China	69.2		
*A.reniforme* var. *reniforme*	?	LPCG002	Cubo de la Galga, La Palma	103.09	97.78 ± 4.06	
		LPCG009	Cubo de la Galga, La Palma	100.88		
		LPCG011	Cubo de la Galga, La Palma	99.42		
		LPCG003	Cubo de la Galga, La Palma	90.45		
		LPCG004	Cubo de la Galga, La Palma	96.77		
		LPCG001	Cubo de la Galga, La Palma	95.92		
		LPCGO14	Cubo de la Galga, La Palma	97.94		
		LPB023	Bermúdec, La Palma	82.18	84.11 ± 2.96	
		LPB006	Bermúdec, La Palma	80.99		
		LPB007	Bermúdec, La Palma	86.83		
		LPB010	Bermúdec, La Palma	86.43		
		TBI001	Barranco del Infierno, Tenerife	95.74	92.75 ± 6.85	
		TBI011	Barranco del Infierno, Tenerife	97.49		
		TBI014	Barranco del Infierno, Tenerife	99		
		TBI017	Barranco del Infierno, Tenerife	88.89		
		TBIO05	Barranco del Infierno, Tenerife	82.64		
		TPH008	Punta del Hidalgo, Tenerife	97.32		
		TPH021	Punta del Hidalgo, Tenerife	93.34		
		TPH010	Punta del Hidalgo, Tenerife	104.57		
		TPH003	Punta del Hidalgo, Tenerife	84.62		
		TPH0017	Punta del Hidalgo, Tenerife	86.86	93.34 ± 8.06	92.92 ± 7.24

### SEM observation

For SEM analysis, mature spores from different populations were dispersed on stubs directly after being collected. The spores were gold-coated in a JFC-1600 Auto Fine Coater and observed using a JEOL JSM-6360LV Scanning Electron Microscope at 25 kV at the South China Botanical Garden, Chinese Academy of Sciences. The spore mean sizes of 7 populations of *A. reniforme* var*. sinense* and 7 populations of *A. reniforme* var. *reniforme* were measured by Smile View software (20 spores per population), and a scatter diagram was made with SPSS. The descriptive terminology in *Spores of Polypodiales (Filicales) from China* [11] and *Plant identification terminology: An illustrated glossary* [37] was followed.

## Results

### Phylogenetic and molecular divergence time analyses

**Figure 2:**
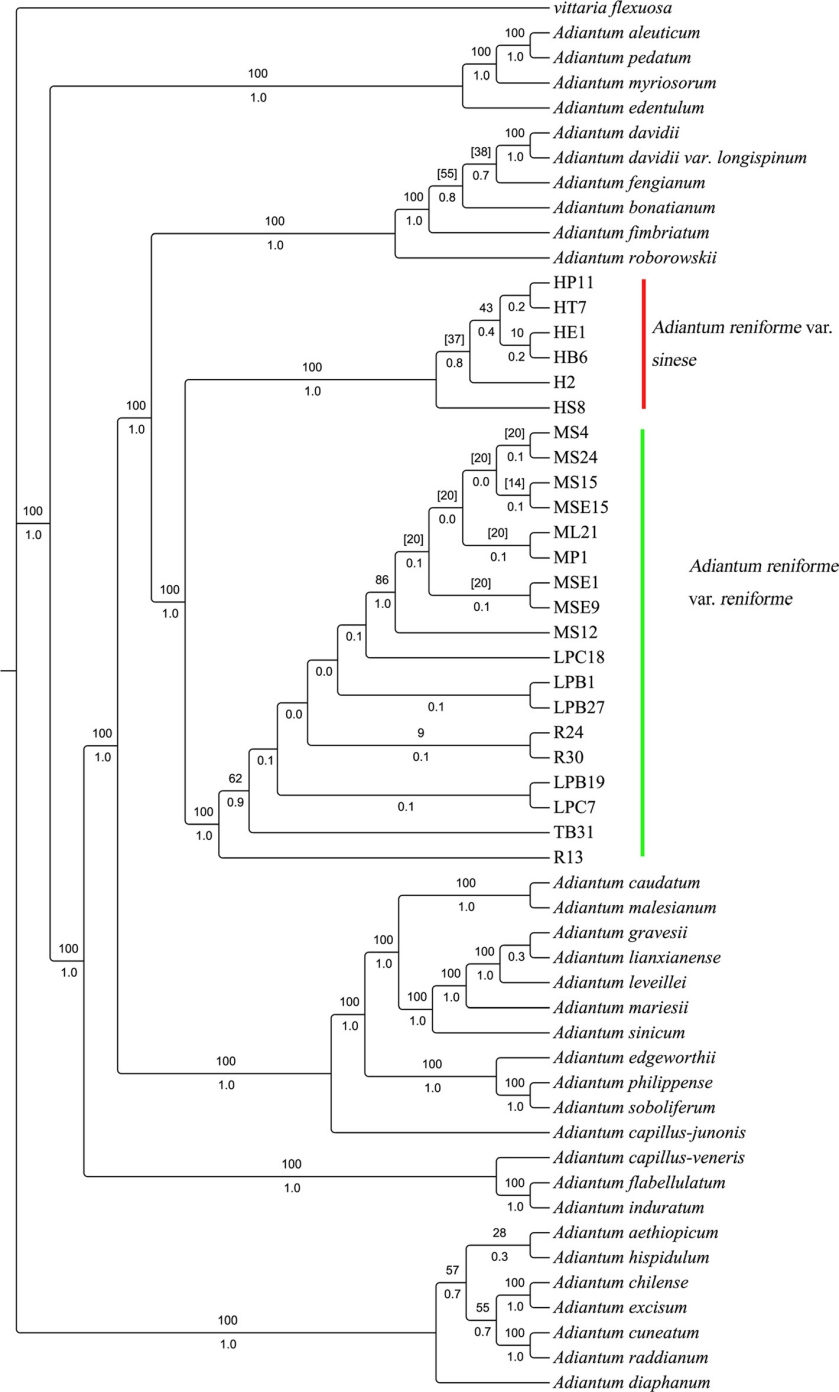
**Strict consensus tree of two maximally parsimonious trees derived from the analysis of the plastid atpA, atpB, trnL-F, and rps4-trnS sequences** **(tree length = 3,911 steps, CI = 0.6423, and RI = 0.8944).** The bootstrap values for 1,000 replicates are shown above the lines, and the Bayesian posterior probabilities are shown below the lines. Front alphabets of HP11, HT7, R13 are the short names of different populations of these two taxa, and the latter numbers represent single individuals.

The topologies derived from analyses of the individual datasets were similar to those obtained from the combined data. Therefore, we emphasised the results of the combined data. The sequences of 23 Chinese species and 8 foreign species of *Adiantum* and *Vittaria flexuosa* (outgroup) were downloaded from GenBank. The combined 4-marker (*atp*A, *atp*B, *trn*L-F and *rps*4-*trn*S) dataset included 56 taxa and consisted of 5,210 nucleotides, of which 1961 were variable (37.6%) and 1,468 were phylogenetically informative (28.2%). Rooted with the specified outgroup *Vittaria flexuosa*, the MP analysis on the combined 4-marker dataset yielded one maximally parsimonious tree of 3,911 steps, a consistency index (CI) of 0.6423, and a retention index (RI) of 0.8944. The tree obtained from the BI analyses had similar topology as the MP strict consensus tree (Figure 2).

All individuals of *A. reniforme* var. *sinense* from different populations were clustered into one clade, and all individuals of *A. reniforme* var. *reniforme* from different populations were clustered into another clade (Figure 2). Our analysis strongly supported that Canary Islands and Madeira *A. reniforme* var. *reniforme* was sister to Chinese *A. reniforme* var. *sinense* (1.0/100). The genetic distance (GD) between *A. reniforme* var. *reniforme* and *A. reniforme* var. *sinense* was calculated by constructing NJ trees using Mega5.0 based on the combined 4-marker data. Compared with the GD between *A. caudatum* and *A. malesianum* (GD = 0.004 ± 0.001) and the distance between *A. flabellulatum* and *A. induratum* (GD = 0.008 ± 0.002), the value between *A. reniforme* var. *reniforme* and *A. reniforme* var. *sinense* (GD = 0.011 ± 0.003) was much longer.

**Figure 3:**
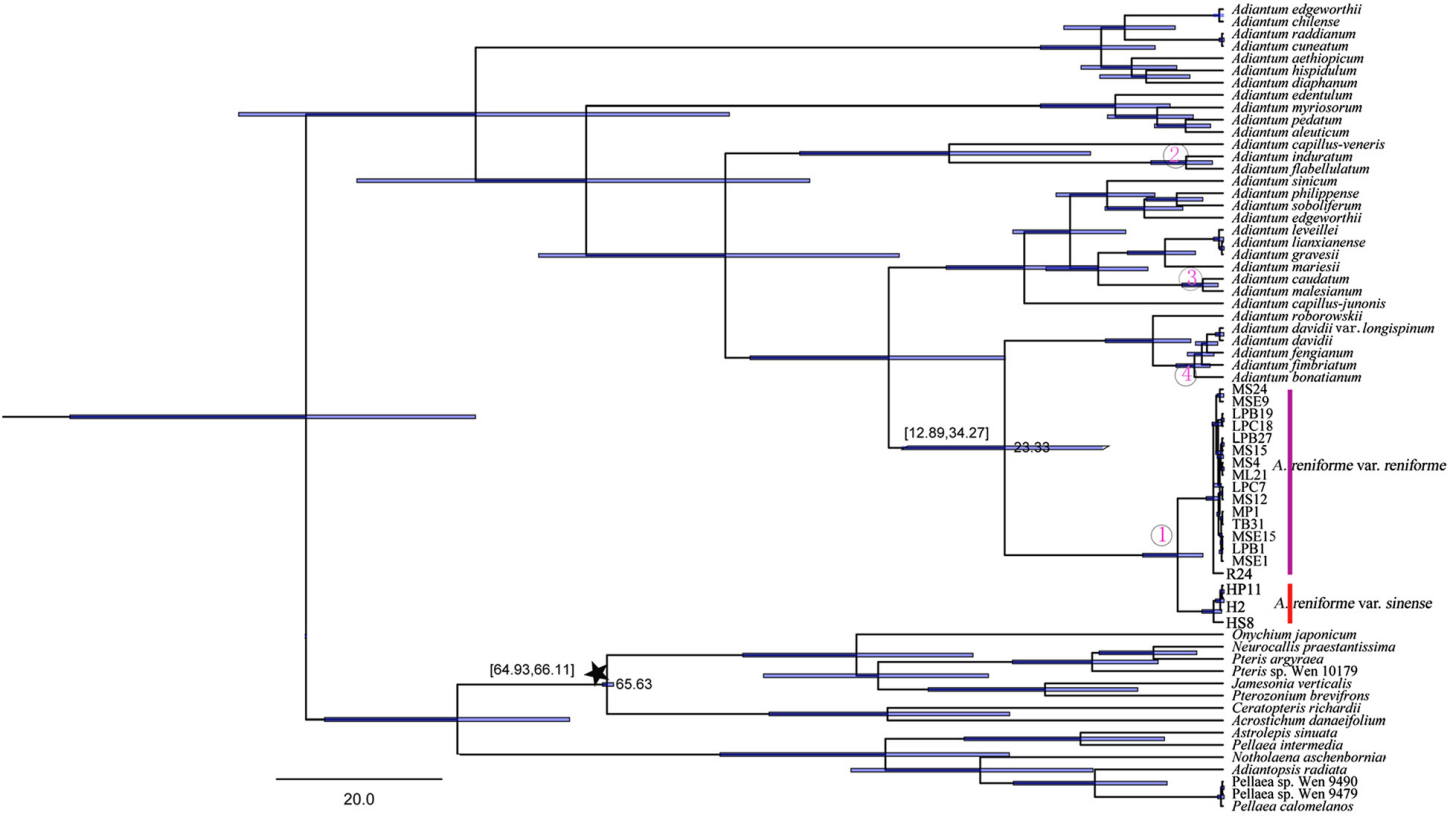
**Chronogram of** ***Adiantum*** **inferred from BEAST with combined sequences (** ***atp*** **A,** ***atp*** **B and** ***rbc*** **L).** The calibration scheme is indicated with black asterisks. Node 1: *A. reniforme* var. *reniforme* and *A. reniforme* var. *sinense*; Node 2: *A. flabellulatum* and *A. induratum*.

The divergence between *A. reniforme* var. *reniforme* and *A. reniforme* var. *sinense* was estimated to be 4.94 (2.26-8.66) Myr, while *A. flabellulatum* and *A. induratum* were dated to diverge 4.06 (1.25-7.80) Myr ago (see Figure 3).

### Chromosome analysis

**Figure 4:**
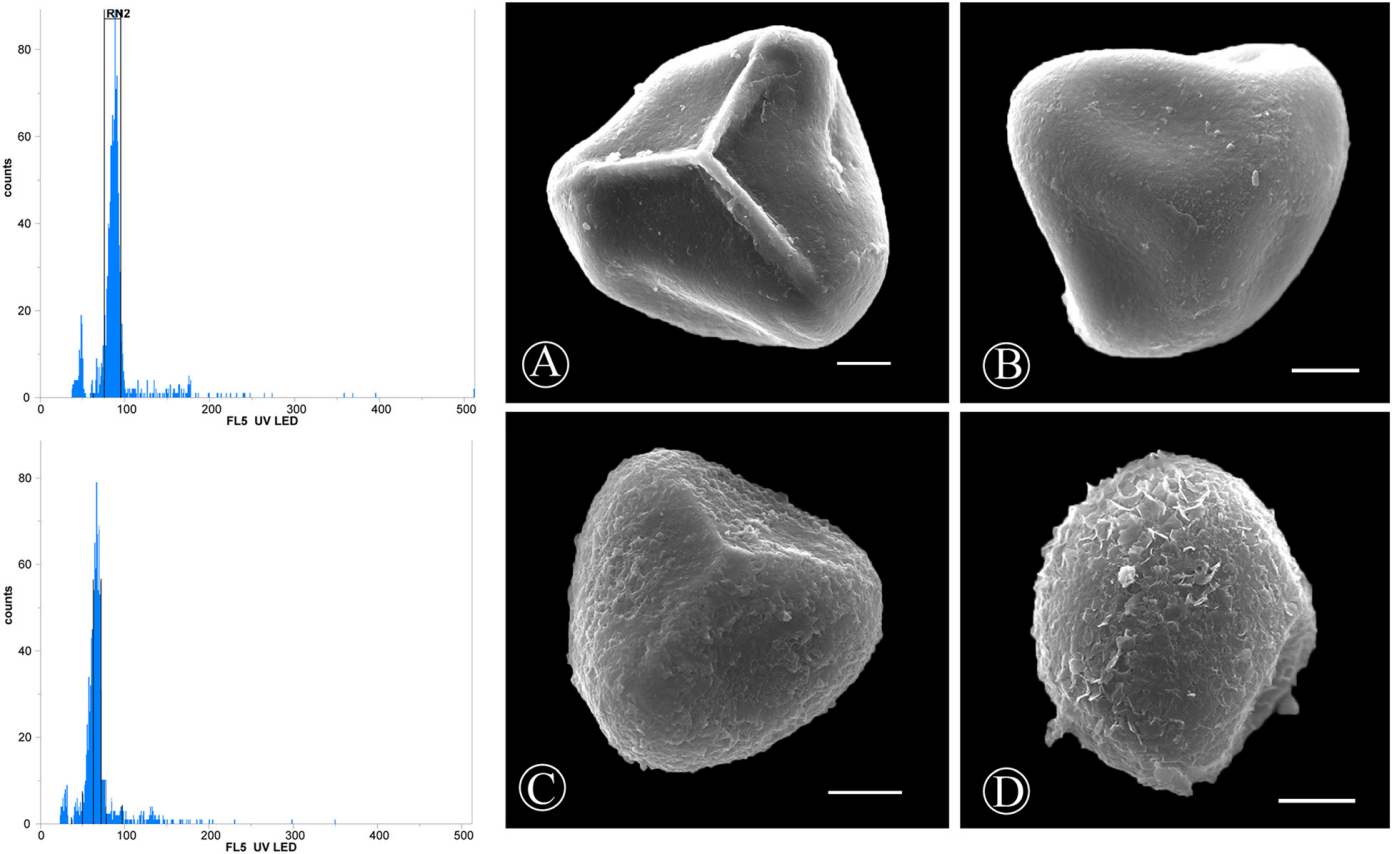
**Flow Cytometric Histogram and SEM Observation of** ***A. reniforme*** **var.** ***reniforme*** **and** ***A. reniforme*** **var.** ***sinense*** **. A** and **C**: proximal surface; **B** and **D**: distal surface.

The ploidy level of *A.reniforme* var. *reniforme* was estimated by comparison with the known tetraploidy *A. reniforme* var. *sinense*. Based on DAPI staining, 21 accessions of *A. reniforme* var. *reniforme* showed relative fluorescence intensities of 92.92 ± 7.24, and 3 accessions of the internal standard *A. reniforme* var. *sinense* showed relative fluorescence intensities of 65.44 ± 3.59 (Table 1). We deduced that *A. reniforme* var. *reniforme* was hexaploidy with 2n = 180, X = 30 because the relative fluorescence intensity of the *A. reniforme* var. *reniforme* accessions was approximately 1.5-fold higher than the *A. reniforme* var. *sinense* accessions. The chromosome number of *A. reniforme* var. *sinense* was determined to be 2n = 120, X = 30 [10]. The flow cytometry histograms of both plants are shown in Figure 4 (left).

### SEM observation and morphological character differences

The spore shapes of both taxa are tetrahedric and are similar in polar and equatorial views. However, the spores are clearly different with respect to surface ornamentation. The spores are actinomorphic and trilete with polar surface triangles, and the equatorial surface is semicircular or super-semicircular. The surface ornamentation of *A. reniforme* var. *reniforme* is psilate, while that of *A. reniforme* var*. sinense* is rugate (see Figure 4). The mean sizes of 7 populations of *A. reniforme* var*. sinense* were 37.1 ± 3.7 μm, which is shorter than the 7 populations of *A. reniforme* var. *reniforme* (47.8 ± 3.9 μm). The spore equatorial axis sizes of *Adiantum* vary from 32 to 55 μm [11], and our findings are consistent with these data.

The morphological characters of these two taxa are obviously different. The leaf blades of *A. reniforme* var. *sinense* are membranous and reniform. Each blade has several concentric rings and yellowish-brown scales. The leaves of *A. reniforme* var. *reniforme* are pachyphyllous and coriaceous and are much rounder and similar to palm. The leaves lack any concentric rings and have deep brown scales (see Figure 1).

## Discussion

### Relationship between *A. reniforme* var. *reniform* and *A. reniforme* var. *asariforme*

The Canary Islands *A. reniforme* var. *reniforme* was determined to be hexaploid in this study based on flow cytometric analyses of sporophytic material. An additional experiment was performed to determine chromosome numbers with conventional squashes of root tip cells but failed because of the huge numbers and crowded chromosomes. Thus, the chromosomes could not be counted using light microscopy.

The ploidy level of *A. reniforme* var. *reniforme* is the same as *A. reniforme* var. *asariforme* if the description in *Flora Republicae Popularis Sinicae*, 3(1) [5] is correct. According to *Flora Republicae Popularis Sinicae*, 3(1), *A. reniforme* var. *asariforme* is another variety of *A. reniforme* var. *reniforme* and is only distributed in South Africa, Madagascar, and Mauritius. Its pachyphyllous and coriaceous leaves have deep brown scales that contain tight and slender white hairs on both surfaces of leaves. The taller and stronger plant size and its hexaploidy are considered the major differences from *A. reniforme* var. *sinense*. However, taller and stronger plants of *A. reniforme* var. *reniforme* are found in fields in La Palma. Its leaves are also pachyphyllous and coriaceous and have deep brown scales. The leaf shape is very similar to the leaf of *A. reniforme* var. *asariforme* based on comparisons of their respective specimens. Therefore, it is reasonable that researchers have treated *A. reniforme* var. *asariforme* as a variety of *A. reniforme* var. *reniforme* [38]. Tardieu-Blot claimed that *A. reniforme* var. *asariforme* was conspecific with *A. reniforme* var. *reniforme* [20]. Further evidence is required to clearly define the relationship between these two varieties.

### Evolution of intercontinental disjunctions between Chinese *A. reniforme* var. *sinense* and Canary *A. reniforme* var. *reniforme*

Three issues have to be discussed to explain the evolution of China-Madagascar-Canary intercontinental disjunctions. The first issue is the original centre of these three taxa. Second, how did the spores spread between each location? Finally, what is the genesis evolution and phylogenetic status of ser. *Reniformia* in *Adiantum* and Pteridaceae?

There are three probable original centres: China; Madagascar or South Africa; the Canary Islands or the western Mediterranean. According to our phylogenetic analysis and molecular divergence estimation results, China is speculated to be the most probable centre. There is strong evidence showing that Chinese *A. reniforme* var. *sinense* is sister to Canary *A. reniforme* var. *reniforme* (BP100; PP1.0; Figure 3). Clades of these two species together form ser. *Reniformia* [5], which has morphological synapomorphies of simple and kidney-shaped blades and clustered short-creeping rhizomes. Ser. *Reniformia* is suggested to be monophyletic and is sister to Ser. *Venusta* (Figure 3), which consists of 10 species and 4 varieties only distributed in Chinese temperate regions. The divergence between *A. reniforme* var. *reniforme* and *A. reniforme* var. *sinense* was estimated to be 4.94 (2.26-8.66) Myr in the Pliocene, and ser. *Reniformia* and Ser. *Venusta* was estimated to diverge in 23.33 (12.89-34.27) Myr in the Miocene. These results indicated that Ser. *Reniformia* and Ser. *Venusta* had a common ancestor at least 23.33 Myr ago but diverged later. The divergence may be related to the intense uplift of the Qinghai-Tibet plateau in the Neocene [39]. The average altitude of the Qinghai-Tibet plateau may have reached 2000 m at 22 Myr [40], during which the landform diversity of the Qinghai-Tibet plateau and climate aridification may have led to the divergence of ser. *Reniformia* from Ser. *Venusta* in China. The Himalayas uplifted rapidly 5.4-2.7 Myr [41], and *A. reniforme* var. *reniforme* diverged from *A. reniforme* var. *sinense* 4.94 (2.26-8.66) Myr. These results indicate that the divergence of the two species may be closely related to the rapid uplift of the Himalayas. Paleomonsoon had existed in China in the Eogene and intensified with the uplift of the Qinghai-Tibet plateau in the Neocene [42]. North-western Eurasia high pressure centres have passed through Southeast Asian nations such as China and India to the Indian Ocean since the Miocene [40,42]. The long distance dispersal of ferns is more common than seed plants because ferns are dispersed by small, windblown spores that are produced in very large numbers and are capable of travelling thousands of kilometres [43-45]. Thus, it was very possible for spores of Chinese *A. reniforme* var. *sinense* to reach the Indian Ocean and Madagascar through winter monsoons and other general atmosphere circulation in winter. Spores of *A. reniforme* var. *sinense* in Madagascar also can get back to China through summer southwest monsoons from the Southern Indian Ocean. However, gene flow was hindered by the high altitude caused by the rapid uplift of the Himalayas in the Pliocene, which caused speciation over time. If China was the origin centre of *A. reniforme*, the dispersal sequence would be as follows: China to Madagascar and then to Canary.

The Canary Islands consist of seven volcanic islands, namely El Hierro, La Palma, La Gomera, Tenerife, Gran Canaria, Fuerteventura, and Lanzarote (from west to east, respectively), located off the north-western African coast. They formed by multiple volcanic episodes [46-48] but showed different evolutionary histories [49]. The western islands of La Palma, El Hierro, and Tenerife are the younger archipelago and are still in their shield stage, which began at most 7.5 Myr ago. The oldest island Fuerteventura began its shield stage 20.6 Myr ago [50]. A fossil of *A. reniforme* var. *reniforme* was discovered in Meximieux near Lyons in the Rhone Valley in Europe [20]. Thus, the Canary Islands may be glacial refugia of *A. reniforme* var. *reniforme* in Quaternary.

## Conclusions

*Adiantum reniforme* var. *sinense* is an independent species rather than a variety of *A. reniforme* var. *reniforme* based on morphological differences, spore observations, chromosome analyses, phylogeny research of the genus *Adiantum* and molecular divergence estimations. Our data are different from Lin YX [1] but in accordance with treatment of Zhang XC [21]. The name *Adiantum nelumboides* X. C. Zhang should be applied to the Chinese taxon as a legal name and the commonly used name for *A. reniforme* var. *sinense* will be treated as a synonym. China is deduced to be the most probable evolution centre of ser. *Reniformia*, and the divergence between *A. reniforme* var. *sinense* and *A. reniforme* var. *reniforme* may be related to the intense uplift of the Qinghai-Tibet plateau in the Neocene. The Canary Islands and Madeira were probably glacial refugia of *A. reniforme* var. *reniforme* in the Quaternary, based on the fossil evidence found in Meximieux near Lyons in the Pliocene.

### Availability of supporting data

The data sets supporting the results of the article are available in GenBank under accession numbers KJ742731-KJ742799 and KJ779969-KJ780019. All of the phylogenetic sequence data in this study are deposited in GenBank (National Center for Biotechnology Information) with the link http://www.ncbi.nlm.nih.gov/nuccore/.

## Additional files

Additional file 1: Table S1. Voucher information and GenBank accession numbers for taxa used in the phylogenetic study on *Adiantum*. Additional file 2: Table S2. Samples examined in the study to estimate divergence times.
