# Supplementary material for: Identification of the relationship between Chinese Adiantum reniforme var. sinense and Canary Adiantum reniforme
Source: BMC Plant Biol. 2015 Feb 5;15:36. doi: 10.1186/s12870-014-0361-9 (PMC4340607; doi:10.1186/s12870-014-0361-9)
Supplement: Additional file 1: Table S1. — Voucher information and GenBank accession numbers for taxa used in the phylogenetic study on Adiantum. [file 12870_2014_361_MOESM1_ESM.pdf]

Additional file 1: Table S1

Voucher information and GenBank accession numbers for taxa used in the phylogenetic study on *Adiantum*. Taxaname, sequence_ID, voucher specimen (herbarium), collection locality, and GenBank accession number in the order of *rbcL*, *atpB*, *atpA*, *trnL-F*, and *rps4-trnS*.

Taxaname	Sequence_ID	Specimen_voucher	GenBank accession number					Collection Locality
			rbcL	atpB	atpA	trnL-F	rps4-trnS	
Adiantum aethiopicum		J. Wen 10780 (US)	JF935350	JF935432	JF937305	JF980695	JF980616	New Zealand
Adiantum aleuticum		Heutte s.n. (US)	JF935362	JF935447	JF937320	JF980709	JF980631	Alaska, USA
Adiantum bonatianum		J.-M. Lu 216 (KUN)	JF935294	JF935371	JF937247	JF980639	JF980556	Yunnan, China
Adiantum capillus-junonis		J.-M. Lu 111 (KUN)	JF935314	JF935395	JF937269	JF980662	JF980578	Guangxi, China
Adiantum capillus-veneris		J. Wen 8192 (US)	JF935332	JF935413	JF937286	JF980676	JF980597	Chongqing, China
Adiantum caudatum		J.-M. Lu 209 (KUN)	JF935296	JF935373	JF937249	JF980641	JF980558	Hainan, China
Adiantum chilense		J. Wen 7313 (US)	JF935336	JF935418	JF937291	JF980681	JF980602	Nuble, Chile
Adiantum cuneatum		J. Wen 10119 (US)	JF935339	JF935421	JF937294	JF980684	JF980605	West Java, Indonesia
Adiantum davidii		J.-M. Lu 344 (KUN)	JF935310	JF935391	JF937265	JF980659	JF980574	Shanxi, China
Adiantum davidii var. *longispinum*		J.-M. Lu 247 (KUN)	JF935292	JF935369	JF937245	JF980638	JF980554	Yunnan, China
Adiantum diaphanum		J. Wen 10727 (US)	JF935301	JF935439	JF937312	JF980702	JF980623	Indonesia
Adiantum edentulum		J.-M. Lu 222 (KUN)	JF935291	JF935368	JF937244	JF980637	JF980553	Yunnan, China
Adiantum edgeworthii		J.-M. Lu 114 (KUN)	JF935311	JF935392	JF937266	JF980660	JF980575	Guangxi, China
Adiantum excisum		J. Wen 7326 (US)	JF935311	JF935419	JF937292	JF980682	JF980603	Concepcion, chile
Adiantum fengianum		J.-M. Lu 228 (KUN)	JF935308	JF935388	JF937262	JF980656	JF980571	Yunnan, China
Adiantum fimbriatum		J.-M. Lu 215 (KUN)	JF935321	JF935402	JF937275		JF980585	Yunnan, China
Adiantum flabellulatum		J. Wen 6585 (US)	JF935325	JF935406	JF937279	JF980670	JF980589	Hainan, China
Adiantum gravesii		J.-M. Lu 451 (KUN)	JF935317	JF935398	JF937272	JF980664	JF980581	Guangdong, China
Adiantum hispidulum		J. Wen 10771 (US)	JF935349	JF935431	JF937304	JF980694	JF980615	Virginia cultivated, USA
Adiantum induratum	6	Hrr_001 (IBSC)	JF935309	KJ742755	KJ742798	KJ779992	KJ779995	Hainan, China
Adiantum leveillei		J.-M. Lu 163 (KUN)	JF935313	JF935394	JF937268	JF980661	JF980577	Guangxi, China
Adiantum lianxianense		J.-M. Lu 441 (KUN)	JF935306	JF935385	JF937259	JF980653	JF980569	Guangdong, China
Adiantum malesianum	10	Hrr_002 (IBSC)	JF935297	KJ742756	KJ742799	KJ779993	KJ779994	Guangzhou, Guangdong, China
Adiantum mariesii		J.-M. Lu 120 (KUN)	JF935302	JF935380	JF937255	JF980648	JF980564	Guangxi, China
Adiantum myriosorum		J.-M. Lu 297 (KUN)	JF935359	JF935444	JF937317	JF980706	JF980628	Chongqing, China
Adiantum pedatum		J.-M. Lu 343 (KUN)	JF935360	JF935445	JF937318	JF980707	JF980629	Shanxi, China
Adiantum philippense		J. Wen 8257 (US)	JF935330	JF935412	JF937285	JF980675	JF980595	Luzon, the Philippines
Adiantum raddianum		J. Wen 9521 (US)	JF935323	JF935404	JF937277	JF980668	JF980587	Antsiranana, Madagascar
Adiantum reniforme	R13	F.W. Xing et F. G. Wang 011 (IBSC)		KJ742737	KJ742780	KJ779975	KJ780002	Barranco del Infierno, Tenerife, Canary Islands, Spain
Adiantum reniforme	R24	F.W. Xing et F. G. Wang 011 (IBSC)		KJ742738	KJ742781		KJ780003	Barranco del Infierno, Tenerife, Canary Islands, Spain
Adiantum reniforme	R30	F.W. Xing et F. G. Wang 011 (IBSC)		KJ742739	KJ742782	KJ779976	KJ780004	Barranco del Infierno, Tenerife, Canary Islands, Spain
Adiantum reniforme	TB31	F.W. Xing et F. G. Wang 011 (IBSC)	KJ742772	KJ742740	KJ742783	KJ779977	KJ780005	Barranco del Infierno, Tenerife, Canary Islands, Spain
Adiantum reniforme	LPB1	F.W. Xing et F. G. Wang 012 (IBSC)	KJ742766	KJ742741	KJ742784	KJ779985	KJ780006	Bermudez, La Palma, Canary Islands, Spain
Adiantum reniforme	LPB19	F.W. Xing et F. G. Wang 012 (IBSC)	KJ742765	KJ742742	KJ742785	KJ779986	KJ780007	Bermudez, La Palma, Canary Islands, Spain
Adiantum reniforme	LPB27	F.W. Xing et F. G. Wang 012 (IBSC)	KJ742770	KJ742743	KJ742786	KJ779987	KJ780008	Bermudez, La Palma, Canary Islands, Spain
Adiantum reniforme	LPC7	F.W. Xing et F. G. Wang 013 (IBSC)	KJ742769	KJ742744	KJ742787	KJ779988	KJ780009	Cubo dela Galga, La Palma, Canary Islands, Spain
Adiantum reniforme	LPC18	F.W. Xing et F. G. Wang 013 (IBSC)	KJ742767	KJ742745	KJ742788	KJ779989	KJ780010	Cubo dela Galga, La Palma, Canary Islands, Spain

Adiantum reniforme	MS4	F.W. Xing et F. G. Wang 017 (IBSC)	KJ742761	KJ742746	KJ742791	KJ779978	KJ780013	St. Vincent, Madeira, Portugal,
Adiantum reniforme	MS12	F.W. Xing et F. G. Wang 017 (IBSC)	KJ742759	KJ742747	KJ742792	KJ779979	KJ780014	St. Vincent, Madeira, Portugal
Adiantum reniforme	MS15	F.W. Xing et F. G. Wang 017 (IBSC)	KJ742760	KJ742748	KJ742793	KJ779980	KJ780015	St. Vincent, Madeira, Portugal
Adiantum reniforme	MS24	F.W. Xing et F. G. Wang 017 (IBSC)	KJ742762	KJ742749	KJ742794	KJ779981	KJ780016	St. Vincent, Madeira, Portugal
Adiantum reniforme	MSE1	F.W. Xing et F. G. Wang 018 (IBSC)	KJ742764	KJ742750	KJ742795	KJ779982	KJ780017	Sexial, Madeira, Portugal
Adiantum reniforme	MSE9	F.W. Xing et F. G. Wang 018 (IBSC)	KJ742771	KJ742751	KJ742796	KJ779983	KJ780018	Sexia, Madeiral, Portugal
Adiantum reniforme	MSE15	F.W. Xing et F. G. Wang 018 (IBSC)	KJ742768	KJ742752	KJ742797	KJ779984	KJ780019	Sexial, Madeira, Portugal
Adiantum reniforme	ML21	F.W. Xing et F. G. Wang 016 (IBSC)	KJ742773	KJ742753	KJ742790	KJ779991	KJ780012	Lugar de Serra, Madeira, Portugal
Adiantum reniforme	MP1	F.W. Xing et F. G. Wang 014 (IBSC)	KJ742763	KJ742754	KJ742789	KJ779990	KJ780011	Pau Bastiao, Madeira, Portugal
Adiantum reniforme var. *sinense*	H2	Wah009 (IBSC)	KJ742757	KJ742731	KJ742774	KJ779969	KJ779998	Xinxiang, Wanzhou, Chongqing, China
Adiantum reniforme var. *sinense*	HP11	Wah007 (IBSC)		KJ742732	KJ742775	KJ779970	KJ779999	Xinxiang, Wanzhou, Chongqing, China
Adiantum reniforme var. *sinense*	HT7	Wah005 (IBSC)		KJ742733	KJ742776	KJ779973	KJ779996	Changping, Wanzhou, Chongqing, China
Adiantum reniforme var. *sinense*	HB6	Wah005 (IBSC)		KJ742734	KJ742777	KJ779971	KJ780000	Changping, Wanzhou, Chongqing, China
Adiantum reniforme var. *sinense*	HE1	Wah010 (IBSC)		KJ742735	KJ742778	KJ779972	KJ780001	Wuling, Wanzhou, Chongqing, China
Adiantum reniforme var. *sinense*	HS8	Wah001 (IBSC)	KJ742758	KJ742736	KJ742779	KJ779974	KJ779997	Xituo, Shizhu, Chongqing, China
Adiantum roborowski		J.-M. Lu 279 (KUN)	JF935289	JF935366	JF937242	JF980635	JF980551	Chongqing, China
Adiantum sinicum		J.-M. Lu 269 (KUN)	JF935300	JF935378	JF937253	JF980646	JF980562	Yunnan, China
Adiantum soboliferum		Y.-X. Zhang 009 (KUN)	JF935299	JF935376	JF937252	JF980644	JF980561	Yunnan, China
Vittaria flexuosa		WP1246 (KUN)	JF935357	JF935441	JF937314	JF980703	JF980625	Vietnam
