# Supplementary material for: Identification of the relationship between Chinese Adiantum reniforme var. sinense and Canary Adiantum reniforme
Source: BMC Plant Biol. 2015 Feb 5;15:36. doi: 10.1186/s12870-014-0361-9 (PMC4340607; doi:10.1186/s12870-014-0361-9)
Supplement: Additional file 2: Table S2. — Samples examined in the study to estimate divergence times. [file 12870_2014_361_MOESM2_ESM.pdf]

Additional file 2: Table S2

Samples examined in the study to estimate divergence times.

Taxon: GenBank accessions, *rbcL*, *atpB*, *atpA*.

Acrostichum danaeifolium: EF452129, EF452008, EF452065.

Adiantopsis radiata: EF452131, EF452010, EF452067.

Astrolepis sinuata: EF452141, EF452021, EF452079.

Ceratopteris richardii: AB059585, AY612691, EF452082.

Jamesonia verticalis: EF452155, EF452038, EF452099.

Neurocallis praestantissima: EF452158, EF452042, EF452104.

Notholaena aschenborniana: EF452159, EF452043, EF452105.

Onychium japonicum: U05641, EF452045, EF452107.

Pellaea intermedia: EF452163, EF452047, EF452109.

Pteris argyraea: EF452169, EF452054, EF452117.

Pterozonium brevifrons: EF452175, EF452061, EF452124.

Pellaea calomelanos: JF935346, JF935428, JF937301.

Pteris sp. Wen 10179: JF935342, JF935424, JF937297.

Pellaea sp. Wen 9479: JF935324, JF935405, JF937278.

Pellaea sp. Wen 9490: JF935328, JF935409, JF937282.
